# Metabolic syndrome and cardiovascular risk

**DOI:** 10.4103/1319-1683.71987

**Published:** 2010

**Authors:** Abdullah M. Alshehri

**Affiliations:** *Department of Internal Medicine, King Fahd Hospital of the University, Al-Khobar, Kingdom of Saudi Arabia*

**Keywords:** Metabolic syndrome, risk, cardiovascular

## Abstract

The constellation of dyslipidemia (hypertriglyceridemia and low levels of high-density lipoprotein cholesterol), elevated blood pressure, impaired glucose tolerance, and central obesity is now classified as metabolic syndrome, also called syndrome X. In the past few years, several expert groups have attempted to set forth simple diagnostic criteria for use in clinical practice to identify patients who manifest the multiple components of the metabolic syndrome. These criteria have varied somewhat in specific elements, but in general, they include a combination of multiple and metabolic risk factors. The most widely recognized of the metabolic risk factors are atherogenic dyslipidemia, elevated blood pressure, and elevated plasma glucose. Individuals with these characteristics, commonly manifest a prothrombotic state as well as and a proinflammatory state. Atherogenic dyslipidemia consists of an aggregation of lipoprotein abnormalities including elevated serum triglyceride and apolipoprotein B (apoB), increased small LDL particles, and a reduced level of HDL cholesterol (HDL-C). The metabolic syndrome is often referred to as if it were a discrete entity with a single cause. Available data suggest that it truly is a syndrome, ie, a grouping of atherosclerotic cardiovascular disease (ASCVD) risk factors, that probably has more than one cause. Regardless of cause, the syndrome identifies individuals at an elevated risk for ASCVD. The magnitude of the increased risk can vary according to the components of the syndrome present as well as the other, non–metabolic syndrome risk factors in a particular person.

## INTRODUCTION

**Figure 1:**
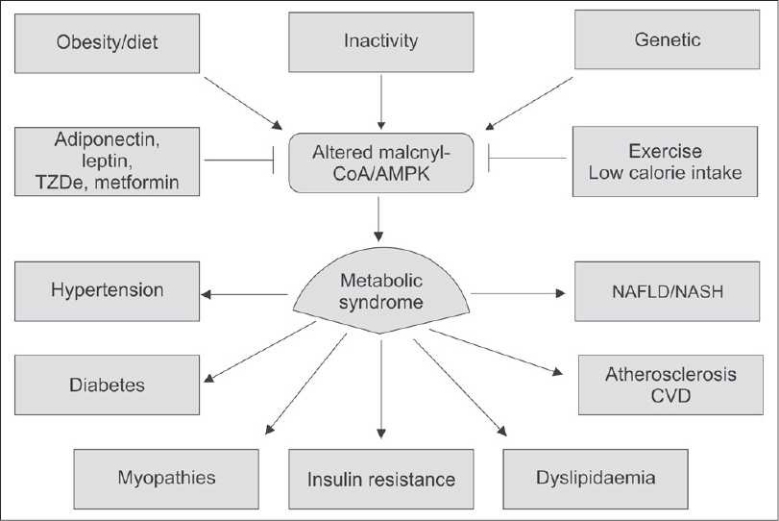
Possible causes and the clinical complications of metabolic syndrome

The metabolic syndrome has become one of the most important topics for this decade because of the marked increase in cardiovascular risk associated with a clustering of risk factors. It is unclear if this truly is a syndrome. What is very clear though, is its clinical implications [Figure 1].

The risk factors associated with this syndrome are primarily well known - hypertension, dyslipidemia (high triglycerides and lower HDL), elevated fasting blood glucose and central obesity. Currently, the American Heart Association classify patients as having this syndrome if they have three out of five abnormal findings.

In a recent Framingham Offspring Study- an analysis tracking more than 3,000 persons over more than a dozen years-prevalence of the metabolic syndrome as defined by Adult Treatment Panel III criteria rose more than 70%. This is of great concern when one considers the associations with type 2 diabetes, more than 80% of whom having metabolic syndrome.

Insulin resistance is strongly associated with most of the risk factors linked with the metabolic syndrome

## PATHOPHYSIOLOGY

The predominant underlying risk factors of the metabolic syndrome appear to be abdominal obesity[1] and insulin resistance;[2] other associated conditions can be physical inactivity,[3] aging[4] and hormonal imbalance.[5] An atherogenic diet (e.g., a diet rich in saturated fat and cholesterol) although not listed specifically as an underlying risk factor for the condition can enhance risk in people with the syndrome for developing cardiovascular disease. One theory states that insulin resistance is the essential cause of metabolic syndrome.[6] There is no doubt that insulin resistance predisposes to the hyperglycemia of type 2 diabetes mellitus. Multiple metabolic pathways have also been proposed to link insulin resistance and compensatory hyperinsulinemia to the other metabolic risk factors.[7] It is recognized that there are some people who though not obese by traditional measures are nevertheless insulin resistant and therefore, have abnormal levels of metabolic risk factors. Examples are seen in individuals with 2 diabetic parents or 1 parent and a first- or second-degree relative who is diabetic;[8] the same is true for many individuals of South Asian ethnic origins.[9] Although insulin-resistant individuals need not be clinically obese, they nevertheless commonly have an abnormal fat distribution that is characterized by predominant upper body fat. Many investigators claim that excess visceral fat is more strongly associated with insulin resistance than any other adipose tissue compartment;[10] other researchers have found that excess subcutaneous abdominal (or truncal) fat also carries a significant association with insulin resistance.[11,12] An interesting feature of upper-body obesity is an unusually high release of nonesterified fatty acids from adipose tissue.[12] This contributes to the accumulation of lipid in sites other than adipose tissue. Ectopic lipid accumulation in muscle and liver seemingly predisposes to insulin resistance[13] and dyslipidemia.[14,15] In obese people adipose tissue is insulin resistant. It raises nonsterified fatty acid levels worsening insulin resistance in muscle[13] and altering hepatic metabolism.[16] In addition, the adipose tissue in obesity exhibits abnormalities in the production of several adipokines that may separately affect insulin resistance and/or modify risk for ASCVD.[16] These include increased production of inflammatory cytokines[17] plasminogen activator inhibitor[18] and other bioactive products.[19] At the same time, the potentially protective adipokine, adiponectin, is reduced.[20] All of these changes have been implicated in the causation of the metabolic risk factors. Indeed, as mentioned before, some individuals exhibit the metabolic syndrome with only a moderate degree of total body obesity.[21] Notable are many South Asians who appear to be inherently insulin resistant,[22] a condition that is exacerbated by mild abdominal obesity. Moreover, the population of the United States varies considerably in degree of insulin resistance.[23] Those with more inherent insulin resistance can develop the metabolic syndrome with only a moderate excess of abdominal fat[21,22] but even people with little or no inherent insulin resistance can develop the metabolic syndrome if they accumulate marked abdominal obesity.[24,25]

Finally, considerable individual and ethnic variation exists in the clinical pattern of metabolic risk factors in obese/insulin-resistant subjects.[26] It is likely that the expression of each metabolic risk factor falls partially under its own genetic control which influences the response to different environmental exposures. For example, a variety of polymorphisms in genes affecting lipoprotein metabolism are associated with worsening of dyslipidemia in obese people.[27] Similarly, a genetic predisposition to defective insulin secretion when combined with insulin resistance can raise plasma glucose to abnormal levels.[28] Although the metabolic syndrome unequivocally predisposes to type 2 diabetes mellitus,[29] many investigators of cardiovascular diseases consider this syndrome a multidimensional risk factor for ASCVD.[1,29] Several recent reports show that the metabolic syndrome is associated with a greater risk for cardiovascular disease[30] with the risk increasing once type 2 diabetes mellitus manifests.[31]

## EPIDEMIOLOGY/PREVALENCE

The prevalence of metabolic syndrome varies by definition and the population studied.[3] Based on data from the third National Health and Nutrition Examination Survey (1988 to 1994), the prevalence of metabolic syndrome (using the NCEP–ATP III criteria) varies from 16 percent of black men to 37 percent of Hispanic women.[3] The prevalence of metabolic syndrome increases with age and increasing body weight.

Alnozhah *et al*. demonstrated in his community-based national epidemiological health survey that the overall age-adjusted prevalence of metabolic syndrome in Saudi Arabia is 39.3% with higher prevalence in females compared to males(42% vs 37.2).[32]

In this study, metabolic syndrome was found to be a risk factor for CAD, whose prevalence was higher among patients with metabolic syndrome (6.7%) compared to subjects without (4.6%)(*P* < 0.0001).

## CLINICAL DIAGNOSIS OF METABOLIC SYNDROME

In the effort to introduce metabolic syndrome into clinical practice, several organizations have attempted to formulate simple criteria for its diagnosis [Table 1]. The first proposal came in 1998 from a group of consultants on the definition of diabetes for the World Health Organization (WHO).[33] This group emphasized that insulin resistance was the major underlying risk factor and required evidence of insulin resistance for its diagnosis. This followed on the widely held belief that insulin resistance was the primary cause of the syndrome. A diagnosis of the syndrome by the WHO criteria could thus be made when a patient exhibited one of several markers of insulin resistance plus two additional risk factors. Although insulin resistance is difficult to measure directly in a clinical setting, several types of indirect evidence were accepted: impaired glucose tolerance (IGT), impaired fasting glucose (IFG), type 2 diabetes mellitus; or impaired disposal of glucose under hyperinsulinemic, euglycemic conditions. The other risk factors used for diagnosis included obesity, hypertension, high triglycerides, reduced HDL-C level, or microalbuminuria. The consultation group suggested categorical cut-off points to define each of these factors. Significantly, the WHO group allowed the term metabolic syndrome to be used for patients with type 2 diabetes mellitus who also met the requirements for the syndrome. They reasoned that patients with type 2 diabetes mellitus often had a clustering of ASCVD risk factors, which put them at particularly high risk for ASCVD.[33] In 1999, the European Group for the Study of Insulin Resistance (EGIR) proposed a modification of the WHO definition.[34] This group used the term insulin resistance syndrome rather than metabolic syndrome. They likewise assumed that insulin resistance was the major cause and required evidence of it for diagnosis. By their criteria, plasma insulin levels in the upper quartile of the population defined insulin resistance. An elevated plasma insulin plus 2 other factors-abdominal obesity, hypertension, elevated triglycerides or reduced HDL-C, and elevated plasma glucose-constituted a diagnosis of insulin-resistance syndrome. Notably, EGIR focused more on abdominal obesity than WHO did, but in contrast to WHO, EGIR excluded patients with type 2 diabetes mellitus from their syndrome because insulin resistance was viewed primarily as a risk factor for diabetes.

**Table 1:** Previous criteria proposed for clinical diagnosis of metabolic syndrome

Clinical Measure	WHO (1998)	EGIR	ATP III (2001)	AACE (2003)	IDF (2005)
Insulin resistance	IGT, IFG, DM-2, or lowered insulin sensitivit* plus any 2 of the following	Plasma insulin _75th percentile plus any 2 of the following	None, but any 3 of the following 5 features	IGT or IFG plus any of the following based on clinical judgment	None
Body weight	Men: waist-to-hip ratio ≥0.90; women: waist-to- hip ratio≥0.85 and/or BMI ≥30 kg/m2	WC≥94 cm in men or ≥80 cm in women	Waist circumference ≥I02 cm in men or ≥88 cm in women	BMI≥25 kg/m2	Increased WC (population specific) plus any 2 of the following
Lipid	TG>150 mg/dL and/ orHDL-C<35 mg/dL in men or <39 mg/DI in women	TG ≥150 mg/dL and/ or HDL-C<35 mg/dL in men or <39mg/dl in women	TG≥150 mg/dL HDL-C_40 mg/dL in men or <50 in women	TG≥150 mg/dL and HDL-C<40 mg/dL in men or<50 mg/	Triglycerides ≥150 mg/dL or on TG Rx HDL-C<40 mg/dL in men or <50 mg/dL in women or on HDL-C Rx
Blood pressure	≥140/90 mm Hg	≥140/90 mm Hg or on hypertension Rx	≥130/85 mm Hg	≥130/85 mm Hg	≥130 mm Hg systolic or 85 mm Hg diastolic or on hypertension Rx
Glucose	IGT, IFG, or T2DM	IGT or IFG (but not diabetes)	≥I10 mg/dL (includes diabetes)	IGT or IFG (but not diabetes)	≥100 mg/dL (includes diabetes)
Other	Microalbuminuria			Other features of insulin resistance	

BMI, body mass index

In 2001, the National Cholesterol Education Program (NCEP) Adult Treatment Panel III (ATP III) introduced alternative clinical criteria for defining the metabolic syndrome.[35] In this case, the purpose of ATP III was to identify people at higher long-term risk for ASCVD who deserved clinical intervention of lifestyle to reduce risk. The ATP III criteria did not require demonstration of insulin resistance per se. It was noted that direct measures of insulin resistance were laborious and not well standardized.

Moreover, less-specific measures, such as glucose tolerance tests, are not routinely used in clinical practice. Although the ATP III panel recognized the phenomenon of clustering of metabolic risk factors, it did not draw conclusions on mechanistic pathogenesis. The ATP III criteria thus required no single factor for diagnosis, but instead made the presence of 3 of 5 factors the basis for establishing the diagnosis. These were abdominal obesity (also highly correlated with insulin resistance), elevated triglycerides, reduced HDL-C, elevated blood pressure, and elevated fasting glucose (IFG or type 2 diabetes mellitus).

In 2005, the International Diabetes Foundation (IDF) published new criteria that again modified the ATP III definition.[36] The IDF writing group included several members of the original WHO group of consultants. They liked the ATP III definition because of its clinical simplicity. Furthermore, they considered that abdominal obesity was so highly correlated with insulin resistance that other more laborious measures of insulin resistance were unnecessary. The IDF clinical definition thus makes the presence of abdominal obesity necessary for diagnosis. When this is present, additional factors originally listed in the ATP III definition are sufficient for diagnosis. IDF recognized and emphasized ethnic differences in the correlation between abdominal obesity and other metabolic syndrome risk factors. For this reason, the criteria of abdominal obesity were specified by nationality or ethnicity based on the best available population estimates. For people of European origin (Europid), the IDF specified thresholds for abdominal obesity to be waist circumferences 94 cm in men and 80 cm in women. These thresholds apply to Europids living in the Americas as well as Europe. For Asian populations, except for Japan, thresholds were 90 cm in men and 80 cm in women; for Japanese they were 85 cm for men and 90 cm for women.

## TREATMENT STRATEGIES

Based on clinical trials, aggressive management of the individual components of the syndrome should make it possible to prevent or delay the onset of diabetes mellitus, hypertension, and cardiovascular disease. All patients diagnosed with metabolic syndrome should be encouraged to change their diet and exercise habits as primary therapy. Weight loss improves all aspects of the metabolic syndrome, besides reducing all-cause and cardiovascular mortality.[37] While many patients find weight loss difficult to achieve, exercise and dietary changes that can lower blood pressure and improve lipid levels will improve insulin resistance, even in the absence of weight loss.[38]

## DIET

No single diet is currently recommended for patients with metabolic syndrome. Therefore, it may be best for physicians to focus on each patient’s specific metabolic alterations when offering dietary advice [Table 2]. Sustained dietary changes may require referral to a registered dietitian to help implement suggestions and ensure adequate micronutrient intake (e.g., calcium, iron, folate) while reducing calories. There is debate about what proportions of macronutrients (i.e., protein, fat, and carbohydrates) produces the best outcome (low-fat, low-carbohydrate, or Mediterranean diets). If a patient is consuming fewer calories than he or she is expending, the macronutrient composition of the diet is probably of secondary importance, because weight loss improves metabolic syndrome.

**Table 2:** AHA/NHLBI criteria for diagnosis of metabolic syndrome

Measure (any 3 of 5 constitute diagnosis of metabolic syndrome)	Categorical cut-off points
Elevated waist circumference	≥102 cm (≥40 inches) in men ≥88 cm (≥35 inches) in women
Elevated triglycerides	150 mg/dL (1.7 mmol/L) or on drug treatment for elevated triglycerides
Reduced HDL-C	≥ 40 mg/dL (≥1.03 mmol/L) in men ≥ 50 mg/dL (≥1.3 mmol/L) in women or on drug treatment for reduced HDL-C
Elevated blood pressure	≥130 mm Hg systolic blood pressure or ≥ 85 mm Hg diastolic blood pressure or on antihypertensive drug treatment in a patient with a history of hypertension
Elevated fasting glucose	≥100 mg/dL Or on drug treatment for elevated glucose

The primary goals of dietary change for metabolic syndrome are to reduce the risk of cardiovascular disease and diabetes mellitus. Two recent Cochrane Database systematic reviews support the role of dietary interventions in helping to reduce cardiovascular risk. Evidence[39] from one large and one small trial showed that a low-sodium diet helps to maintain lower blood pressure following withdrawal of antihypertensive medications. Results from clinical trials of low-fat diets in which participants were involved for more than two years showed significant reductions in the rate of cardiovascular events and suggested protection from total mortality.[40] The degree of protection from cardiovascular events was statistically significant in patients with a higher risk of cardiovascular disease According to the Dietary Approaches to Stop Hypertension (DASH) study[41] patients who consumed a diet low in saturated fat and high in carbohydrates experienced a significant reduction in blood pressure, even without weight reduction. The DASH diet emphasizes fruits, vegetables, low-fat dairy foods, whole grains, poultry, fish, and nuts, while reducing saturated fats, red meat, sweets, and sugar containing beverages. Reducing sodium intake can further reduce blood pressure or prevent the increase in blood pressure that may accompany aging. Low-fat, high-carbohydrate diets have been criticized because they may raise triglyceride levels and lower HDL cholesterol levels in some patients, thus aggravating the dyslipidemia of metabolic syndrome. To treat hypertriglyceridemia, or the decline of HDL-cholesterol levels on a low-fat diet, carbohydrate intake can be reduced and replaced with foods high in monounsaturated fats or low glycemic index carbohydrates. These changes create a diet similar to the Mediterranean-style diet, which also has been shown to reduce mortality from cardiovascular disease.[42]

## EXERCISE

Skeletal muscle is the most insulin-sensitive tissue in the body and, therefore, a primary target for impacting insulin resistance. Physical training has been shown to reduce skeletal muscle lipid levels and insulin resistance, regardless of BMI. The impact of exercise on insulin sensitivity is evident for 24 to 48 hours and disappears within three to five days. Thus, regular physical activity should be a part of any effort to reverse the effects of insulin resistance.

The goal for family physicians is to help patients find a level of activity that they can accomplish over the long term. A combination of resistance and aerobic exercise is best, but any activity is better than none, and patients who have been sedentary need to start with walking and gradually increase duration and intensity, use of low-weight dumbbells, elastic exercise bands, or even heavy food containers can provide the needed weight for resistance training. Walking or light jogging for one hour per day will produce significant loss of abdominal (visceral) fat in men without caloric restriction.[43]

## TREATMENT OF THE INDIVIDUAL COMPONENTS OF THE METABOLIC SYNDROME ATHEROGENIC DYSLIPIDEMIA

### Primary aims for therapy:

- Lower TG (as well as lowering ApoB and non-HDL cholesterol)
- Raise HDL-c levels
- Reduce LDL-c levels (elevated levels represent a high risk in the metabolic syndrome)

#### Options:

- Statins to reduce all ApoB-containing lipoproteins and achieve ATP III goals for LDL-c as well as for non-HDL-C. Several clinical studies have confirmed the benefits of statin therapy.
- Fibrates (PPAR alpha agonists) improve all components of atherogenic dyslipidemia and appear to reduce the risk for CVD in people with metabolic syndrome. The Veterans Affairs High-Density Lipoprotein Intervention Trial (VA-HIT) showed that raising HDL-c concentrations using a fibrate in patients with well-established CHD and both a low HDL-c and a low LDL-c level will significantly reduce the incidence of major coronary events.
- Fibrates in combination with statins but may be complicated by side effects

### Elevated blood pressure

- Categorical hypertension (BP 140/90mmHg) should be treated according to the USA Seventh Report of the Joint National Committee on prevention, detection, evaluation, and treatment of high blood pressure (JNC 7) recommendations.
- In patients with established diabetes, antihypertensive therapy should be introduced at BP130/80mmHg.

#### Options:

- Angiotensin converting enzyme inhibitors and angiotensin receptor blockers are useful drugs, with some clinical trials (but not all) suggesting they carry advantages over other drugs in patients with diabetes. At this time, however, the majority of clinical trials suggest that the risk reduction associated with antihypertensive drugs is the result of blood pressure lowering per se and not due to a particular type of drug.
- No particular agents have been identified as being preferable for hypertensive patients who also have the metabolic syndrome.

## INSULIN RESISTANCE AND HYPERGLYCEMIA

There is growing interest in the possibility that drugs that reduce insulin resistance will delay the onset of type 2 diabetes and will reduce CVD risk when metabolic syndrome is present. The Diabetes Prevention Program (DPP) showed that metformin therapy in patients with prediabetes will prevent or delay the development of diabetes and recent thiazolidinedione studies have also demonstrated efficacy in delaying or preventing type 2 diabetes in patients with impaired glucose tolerance (IGT) and insulin resistance. Similarly, other studies have shown that both acarbose and orlistat can be used to delay the development of type 2 diabetes in patients with IGT. Data do not yet exist to show whether any of the currently available thiazolidinediones reduce the risk of CVD in those with the metabolic syndrome, IGT or diabetes.

## CONCLUSION

Metabolic syndrome is increasingly being recognized as a constellation of clinical criteria that predisposes individuals to a significant cardiovascular risk and the development of Type 2 diabetes. With up to 40% of the Saudi population meeting the criteria for this syndrome and an emerging epidemic of global obesity, clinicians should recognize the syndrome and aggressively manage these individuals with lifestyle modification, education and if necessary, pharmacological intervention.
